# ﻿Description of the larva of *Platycnemisphasmovolans* Hämäläinen, 2003 (Odonata, Platycnemididae), with a key to the larvae of the subfamily Platycnemidinae from the Sino-Japanese and Oriental regions

**DOI:** 10.3897/zookeys.1221.138079

**Published:** 2024-12-10

**Authors:** Tosaphol Saetung Keetapithchayakul, Quoc Toan Phan

**Affiliations:** 1 The Center for Entomology & Parasitology Research, College of Medicine and Pharmacy, Duy Tan University, 120 Hoang Minh Thao, Lien Chieu, Da Nang, Vietnam Duy Tan University Da Nang Vietnam

**Keywords:** Biological notes, Coenagrionoidea, damselfly, identification key, new record, Platycnemidini, Vietnam, Zygoptera

## Abstract

The final instar larva of the rare species *Platycnemisphasmovolans* Hämäläinen, 2003 is described and illustrated here for the first time, including a new distribution record from Vietnam. The larva of *P.phasmovolans* differs from that of congeneric species by distinct morphological features, including the presence of four setae on the palpal lobe of the labium, the presence of lateral spines on abdominal S5–9, and a long terminal filament on the caudal lamella. We also provide a key to species for the known larvae of the subfamily Platycnemidinae in the Sino-Japanese and Oriental regions.

## ﻿Introduction

The genus *Platycnemis* Burmeister, 1839, comprising 11 recognized species (Paulson et al. 2024), belongs to the subfamily Platycnemidinae (tribe Platycnemidini), family Platycnemididae (Dijkstra et al. 2014). This genus is widely distributed across Europe, extending eastward into Asia. The Asian species of *Platycnemis* are represented by four species from East Asia (*P.echigoana* Asahina, 1955; *P.foliacea* Selys, 1886; *P.phyllopoda* Djakonov, 1926; and *P.sasakii* Asahina, 1949) and one species from mainland Southeast Asia (*P.phasmovolans* Hämäläinen, 2003). *Platycnemisphasmovolans*, known for their elusive nature, have been documented in specific locations within central Laos and southern China, suggesting a broader but still undefined range that undoubtedly extends into northern Vietnam (Hämäläinen 2020). The species was first found in the Kaew Neua Pass area, Lak Sao district, Bolikhamsai Province in 2001 and 2002 (Hämäläinen 2003); and later, a small population was rediscovered (by Naoto Yokoi) in 2016 near the type locality (Hämäläinen 2020). While the coordinates provided in Hämäläinen (2011) are generally accurate, the actual location of the type locality is at 18°22'17.35"N, 105°09'05.84"E, which is approximately two kilometers from the Laos-Vietnam border (Hämäläinen pers. comm.). In China, a male specimen was photographed in 2009 in the Maolan Nature Reserve in Guizhou (Pu et al. 2019), and another was recorded in 2018 near Nanning, Guangxi (Hämäläinen 2020).

The larvae of Platycnemidinae display a variety of morphological traits that are essential for species identification and add to our understanding of evolutionary relationships within the subfamily (Dijkstra et al. 2014; Orr and Dow 2015; Saetung et al. 2020). The Asian genera *Platycnemis*, *Pseudocopera* Fraser, 1922 and *Copera* Kirby, 1890, all exhibit distinctive larval morphology. The larva of *Matticnemis* Dijkstra, 2013, a monotypic genus from northern Vietnam, remains unknown. Studying platycnemidid larvae is challenging due to the high degree of morphological similarity among species, with many larvae either undescribed or with inadequate published descriptions (Keetapithchayakul et al. 2022). In this study, we describe the final instar larva of *P.phasmovolans* for the first time. We compare its characteristics with those of other described congeneric species. Additionally, we present new distribution records and provide a key to the known larvae of species of the Platycnemidinae in the Sino-Japanese and Oriental regions.

## ﻿Material and methods

**Figure 1.**
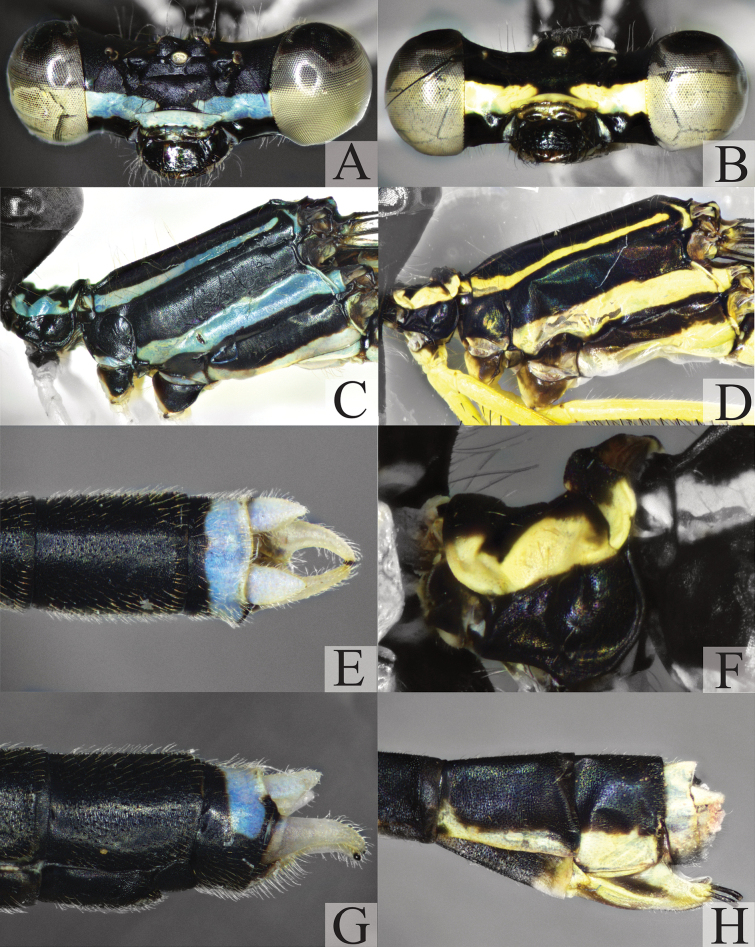
Adult characters of *Platycnemisphasmovolans***A, C, E, G** male **B, D, F, H** female **A, B** head, frontal view **C, D** thorax, lateral view **E** abdominal tip including appendages, dorsal view **F** posterior pronotal lobe of prothorax in lateral view **G–H** abdominal tip including appendages, lateral view.

Final instar larvae were collected from headwater streams of the North Central Region of Vietnam using D-frame nets and sorted manually with sieves. The larvae were transported to the laboratory and reared in plastic containers until they reached adulthood. They were fed *Aedes* larvae and provided with toothpicks as substrates to support emergence. Identification of the emerged adults was based on Hämäläinen (2003) (Figs 1, 8). Measurements and photographs were taken using a ZEISS Stemi 508 stereomicroscope equipped with an OPTIKA C-P6 Digital Camera. Illustrations were created using the Procreate application on an iPad Pro 2020, based on representative digital photographs. Final plates were assembled using AFFINITY Photo 2 version 2.5.3.

The descriptions of the larval mandibular formula, generic characteristics, and distinctive surface features of the larvae follow Watson (1956), Saetung et al. (2020) and Keetapithchayakul et al. (2022), respectively. Specimens are deposited in the Zoological Collection of Duy Tan University (ZCDTU), Da Nang City, Vietnam.

### ﻿Abbreviations used

**S1–10** abdominal segments 1–10

**A1–7** antennomeres 1–7

## ﻿Results

### ﻿ Platycnemis phasmovolans

Taxon classificationAnimaliaOdonataPlatycnemididae

Hämäläinen, 2003

5BF95E02-CDDB-533D-BB9B-52E853D310C3

#### Material examined (larva).

Vietnam • 1 exuviae: 1 ♂ (collected as last stadium larva, reared in laboratory); 27 Jul. 2024; 18°59'24.4"N, 104°50'17.8"E; elevation 266 m a.s.l.; Yen Khe Commune, Con Cuong District, Nghe An Province; T.S. Keetapithchayakul leg.; ZCDTU. • 7 late stadium larvae: 1 ♂ (F-0), 1 ♀ (F-0), 1 ♂ (F-1), 2 ♀♀ (F-1), 1 ♂ (F-2), 1 ♀ (F-2); 27 Jul. 2024; same site and collector as above; ZCDTU. • 2 early stadium larvae: 1 ♂, 1 ♀; 27 Jul. 2024; same site and collector as above; ZCDTU.

#### Description of larva.

**Figure 2.**
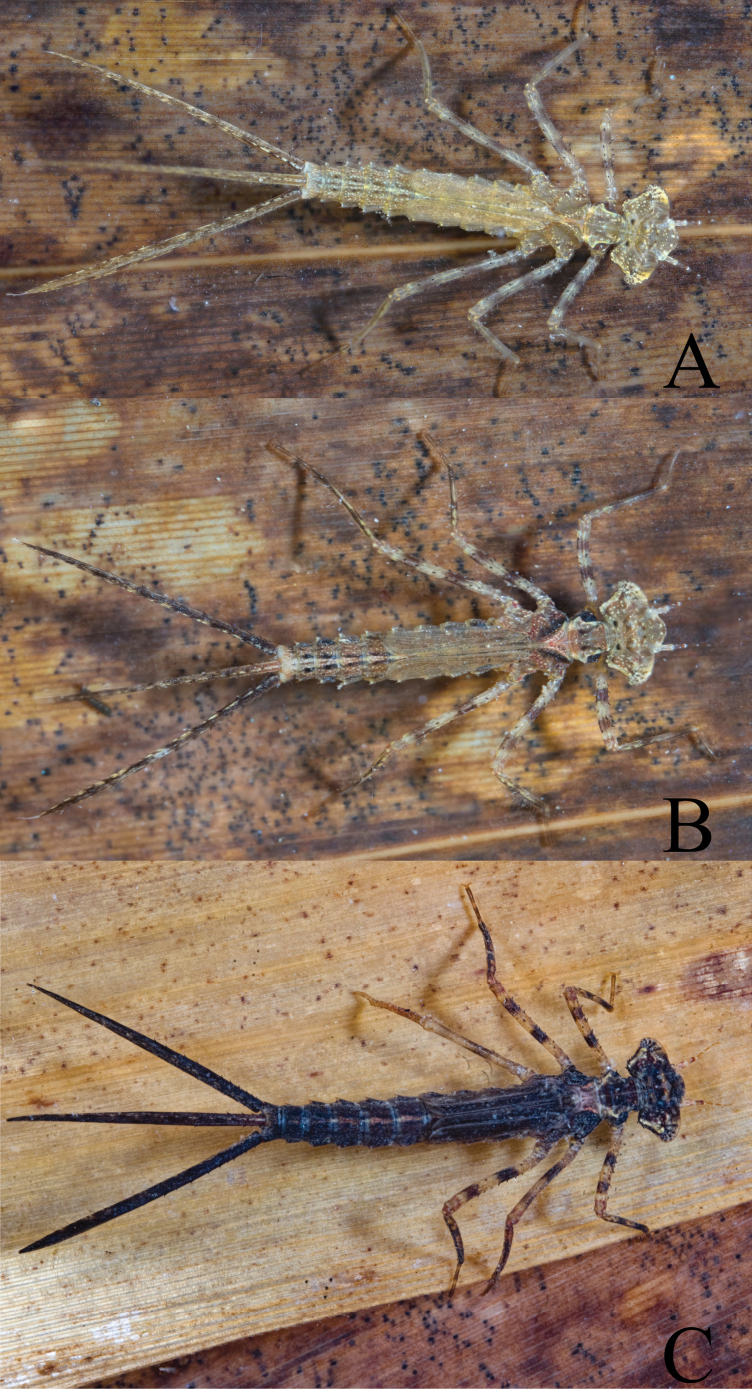
Colour variation of *Platycnemisphasmovolans* larva **A** pale yellowish-brown **B** dark yellowish-brown **C** blackish-brown.

**(based on 1 male (exuviae) and 1 female (F-1))** Habitus (Fig. 2) slender and elongate, long thin legs, abdomen cylindrical, slightly tapered caudad, lamellae of caudal gills with terminal filament at apex; coloration varies from yellowish-brown to bright green brownish to brownish-black.

**Figure 3.**
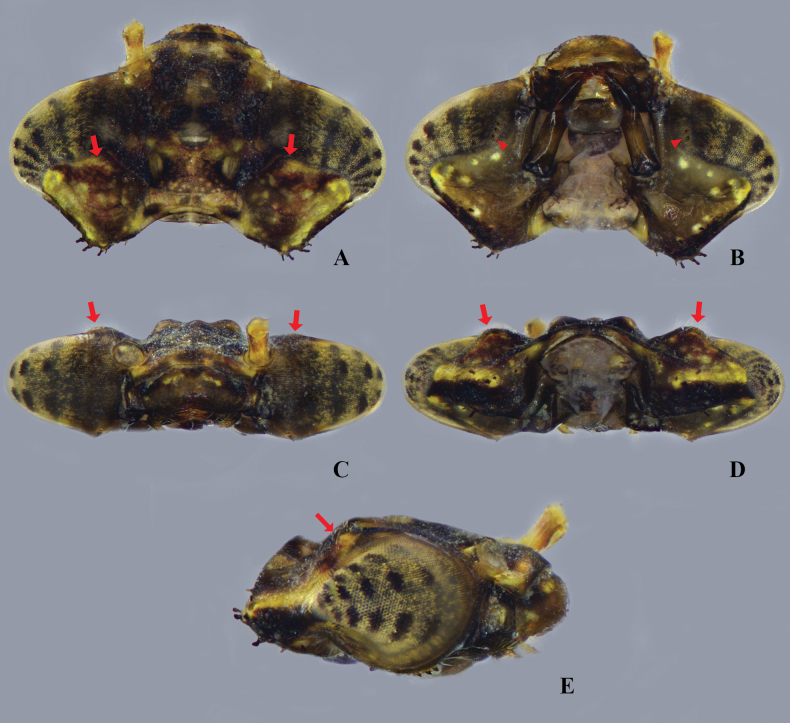
Head of *Platycnemisphasmovolans* larva **A** dorsal view **B** ventral view **C** frontal view **D** back view **E** lateral view. Arrow: prominence on occipital margin; triangle: row of spine and setae on genae.

**Figure 4.**
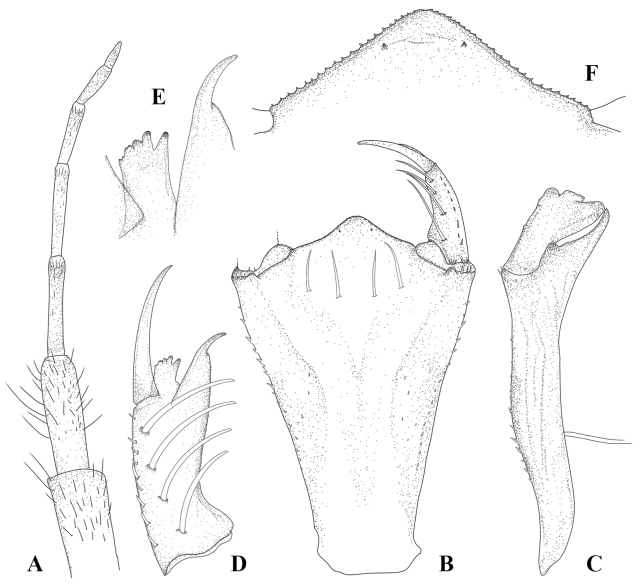
Antenna and mouth parts of *Platycnemisphasmovolans* larva **A** left antenna **B** prementum, dorsal view **C** prementum, lateral view **D** left labial palp **E** detail of distal left palpal lobe **F** ligula (median lobe).

**Figure 5.**
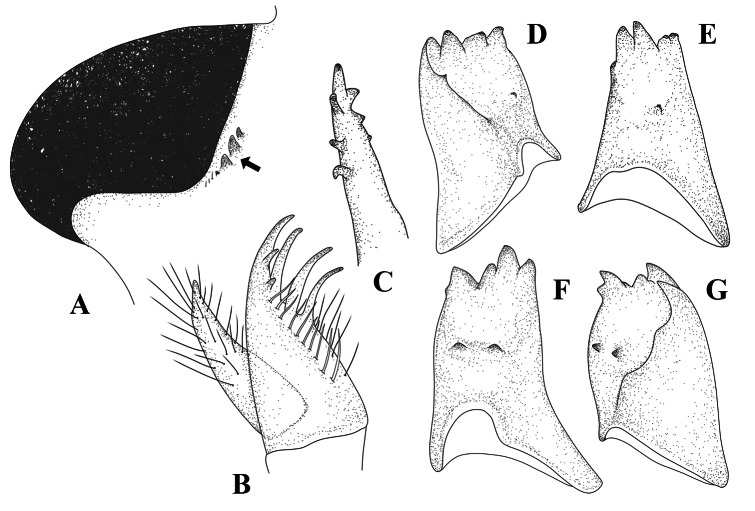
Compound eyes and mouth parts of *Platycnemisphasmovolans* larva **A** genae **B** left maxilla, dorsal view **C** left maxilla, lateral view **D** right mandible, ventro-internal view **E** right mandible, internal view **F** left mandible, internal view **G** left mandible, ventro-internal view. Arrow indicates row of spines and setae.

***Head***: broad in dorsal view, roughly a strongly squashed pentagon in outline, with hind margin strongly excavated; bearing scattered simple setae; labrum flattened ventrally, outline with convex corners flanking central anterior concavity, with minute tubercles on distal half and basal glabrous; frons and vertex strongly raised with prominent ocelli; compound eyes narrow and rounded protruding postero-laterally; occiput with dense minute tubercles and scattered simple setae, convex in outline, anterior occiput with low raised prominences just behind margin of eyes (Fig. 3, indicated by red arrows); postocular lobes, rounded, scattered simple setae on anterior margin, with scattered papilliform setae and robust spiniform setae on posterior margin; genae (Figs 3B, 5A) with row of 2–3 blunt stout spines and simple setae on anterolateral margin. Antennae (Fig. 4A) filiform, 7-segmented with A2 the longest, relative length of antennomeres 0.93: 1 (0.4 mm): 0.95: 0.85: 0.58: 0.4: 0.33. Prementum (Fig. 4B) elongate subpentagonal shape, its basal hinge reaching anterior of mid coxae when mask folded; with two pairs of strong premental setae; lateral margin at base of palp with 3–4 distinct spiniform setae; with a row of 16–18 spiniform setae along distal half of lateral margin, with 1 pair long thin simple setae on middle of ventral side (Fig. 4C); ligula (Fig. 4F) strongly produced to form an obtuse angle, the two sides slightly convex; with one pair of short subapical protuberances and minute spiniform setae along margin; lobe of labial palp (Fig. 4D) 0.41 length of prementum with 4 setae on palpal lobe, outer margin with row of short spiniform setae, inner margin with weakly crenate; apex with 2 processes, the outer one truncate, but with a distinctly slanted or curved margin and bearing 5 distinct teeth, the innermost being largest and most isolated; inner process tapered then abruptly narrowed to thin acutely tipped end hook (Fig. 4E); movable hook slender and about 0.60 times as long as palpal lobe, acuminate, bent slightly inwards. Maxilla (Fig. 5B, C) galeolacinia with 7 teeth, 4 dorsal teeth approximately of the same size, apical teeth largest, 3 ventral teeth of small size. Mandible (Fig. 5D–G) with mandibular formula: L 1+1’234 a b/ R 1+1’234 y a, asymmetrical, robust with well-developed long teeth on each incisor lobe; left mandible with five incisor teeth, two molar teeth (a = b); right mandible with five incisor teeth, one molar tooth, an additional tooth.

***Thorax***: narrower than head, with scattered simple setae, papilliform setae, and white spots. Prothorax dorsally flattened; lateral angles acute, projecting sharply at postero-lateral corners; posterior margin gently keeled midline, forming subtle ridge across posterior border, rounding at lateral edges. Synthorax robust, slightly elongated; mesepisternum with pronounced lateral keels defining boundary with mesepimeron; keels slightly raised, forming well-defined ridge; dorsal surface of mesepisternum with faint longitudinal ridges, aligned parallel to midline; mesinfraepisternum slightly convex; wing pads pale with glabrous, parallel, anterior and posterior wing pads reaching to distal margin of S6; legs almost flat and long; femora thin with dark band on posterior side, row of spiniform setae and scattered simple setae; tibial comb with scattered setae and a few tridentate setae; two claws simple with pulvilliform empodium.

**Figure 6.**
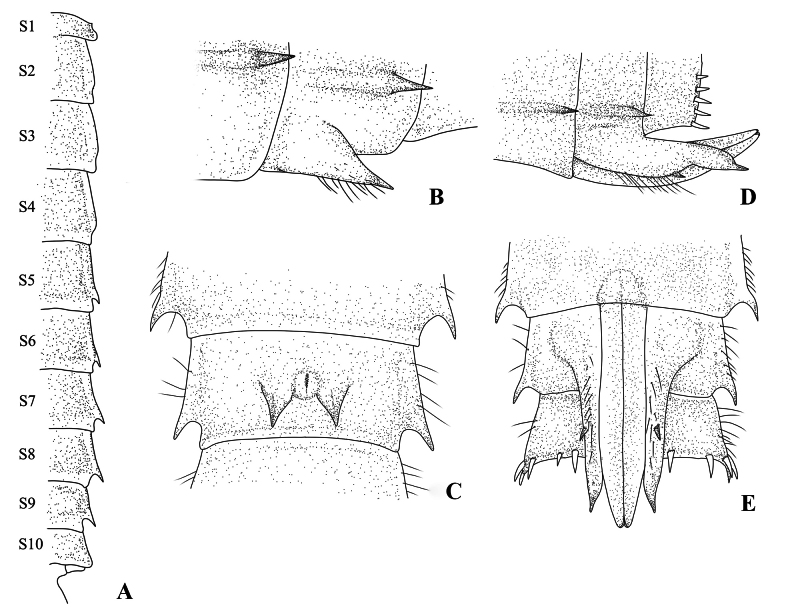
Abdomen and gonapophysis of *Platycnemisphasmovolans* larva **A** S1–10, dorsal view **B** male gonapophysis, lateral view **C** male gonapophysis, dorsal view **D** female gonapophysis, lateral view **E** female gonapophysis, dorsal view.

**Figure 7.**
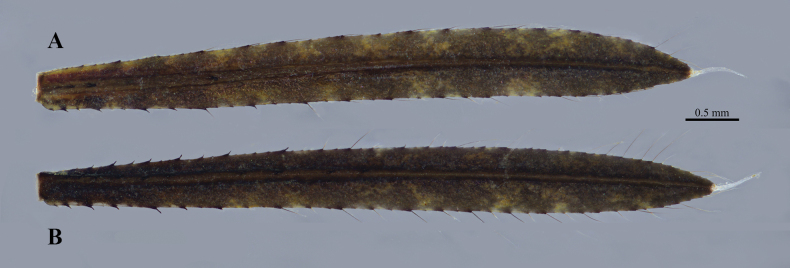
Caudal lamellae of *Platycnemisphasmovolans* larva **A** median lamella **B** lateral lamella.

***Abdomen***: cylindrical, slender, narrowing caudally, scattered simple setae, minute tubercles and white spots; abdominal terga with pale longitudinal line, posterior margin with pair of pale black spots; abdominal sterna smooth; abdominal pleura flatted on S2–S9, with scattered simple setae, and row of spiniform setae and simple setae on lateral margin, lateral spines on abdominal S5–S9 (S9≥S8≥S7>S6>>S5) (Fig. 6A). S10 with cluster of spiniform setae externally at the basal of cerci; male gonapophyses (Fig. 6B, C) broad-based, conical, sharply pointed, slightly divergent in ventral view, almost reaching anterior margin of S10, with a row of simple setae on ventral margin; gonopore small, O-shape embossed with median fissure; female gonapophyses (Fig. 6D, E) with two pairs of long valvae; lateral valvae terminating in sharply pointed processes, slightly divergent, with a distinct postmedian ventral spine preceded by row long simple setae on each side, extending over sternite 10; central valvae longer than lateral valvae, smooth, slender, apically rounded. Caudal lamellae (Fig. 7) long and narrow, lanceolate, with irregular light and dark-brown markings, rather sparse spiniform setae and simple setae of variable lengths along margin, median trachea; terminal filament pale and slim; a distinct median trachea with spiniform setae array on both sides, reaching terminal filament; median lamella only slightly shorter and broader than lateral lamella.

#### Material examined (adult).

Vietnam • 4 ♂♂; 21 May 2024; 18°59'24.4"N, 104°50'17.8"E; elevation 266 m a.s.l.; Yen Khe Commune, Con Cuong District, Nghe An Province; Q.T. Phan leg.; ZCDTU. • 3 ♂♂, 2 ♀♀; 27 Jul. 2024; same site and collector as above.

#### Additional observations (adult).

China • 1 ♂ 26–31 May 2020; 25°18'36.0"N, 107°54'00.0"E; Maolan National Nature Reserve, Guizhou, Libo County, Province, Ruibin Song observer.

#### Brief description of adult.

**Figure 8.**
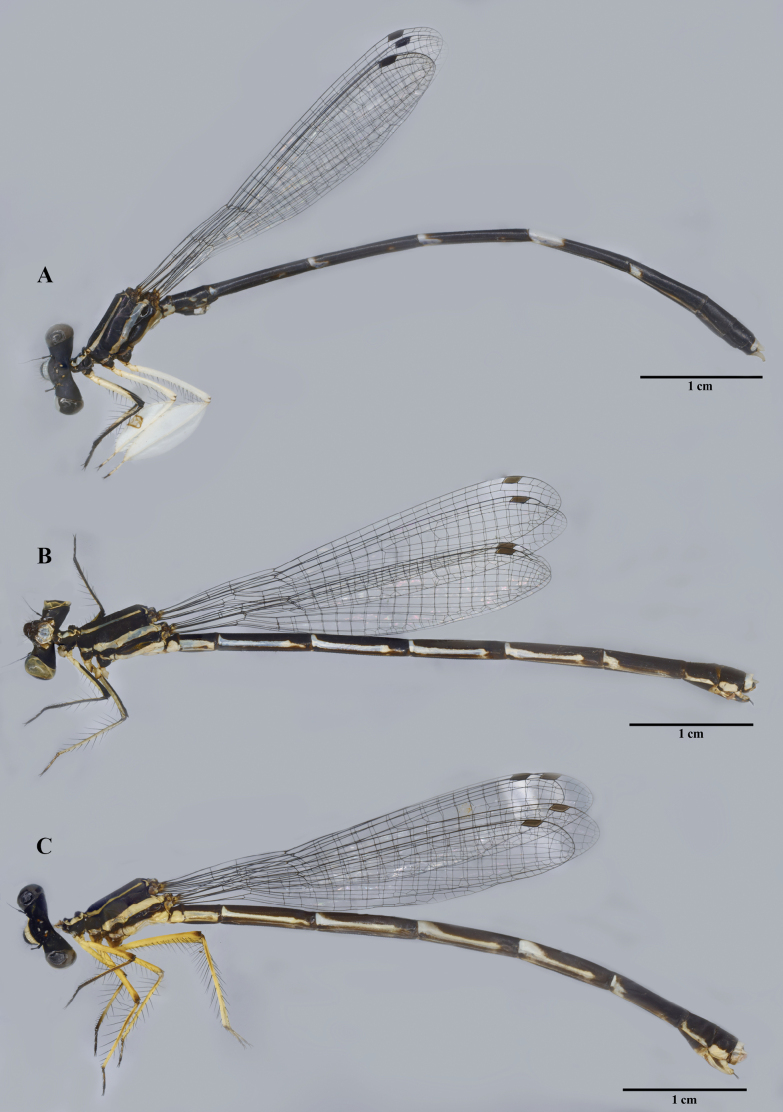
Adult habitus **A** male **B** female **C** immature female.

**Male** (Fig. 8A) – ***Head***: black with broad bluish transverse stripe across frons and genae, broken in middle above pale blue postclypeus (Fig. 1A). ***Thorax***: prothorax black with paired blue lateral markings on pronotum with irregular inner margins; propleuron entirely black (Fig. 1C); synthorax black with narrow blue antehumeral stripes, two distinct lateral stripes: one pale blue on metepisternum, the other tending to very pale blueish-yellow on lower margin of metepimeron (Fig. 1C); fore legs with femora white, except anterior side which is black in the apical third; tibiae and tarsi black; mid and hind legs with femora and tibiae wholly white, tibiae enormously dilated, feather-like, length 2.87 times and 3.22 times maximum width, respectively. *Abdomen*: black with blueish-white pattern: S1 with heart-shaped spot laterally; S2 with narrow stripes along lower margin and 1–2 markings above stripes; S3-6 with basal rings, concave dorsally, smallest on S3 and largest on S6, that on S3 incomplete dorsally; S6 marking one-third length of segment; S7 with ventro-lateral basal marking; S8–9 entirely black; posterior half of S10 dorsum blue to pale yellowish (Fig. 1G); appendages pale, as illustrated (Fig. 1E, G).

**Female** (Fig. 8B, C): As male unless otherwise stated (Fig. 1B, D, F, H): colour pattern blue replaced by yellow tending to white (Fig. 8B, C). Posterior pronotal lobe weakly developed, smooth, without spine. Tibiae not dilated. Abdomen, full stripe on S1, S2–6 the basal bands extend dorsally but do not form complete rings. S7 with on ventro-distal marking, but pale in middle; S8 with complete pale yellow broad ventral border to tergite; S9 broadly pale yellow ventrally, S10 and anal appendages entirely pale (Fig. 1H).

This description agrees almost exactly with Hämäläinen (2003) and hence we may be confident of the identity of the larvae from which specimens were bred.

#### Distribution.

Lao PDR: Bolikhamsai Province, Kaew Neua Pass area (Hämäläinen 2003, 2020); Vietnam: Nghe An Province, Yen Khe Commune, Con Cuong District (this study, new record); China: Guizhou Province, Libo County, Maolan National Nature Reserve (Pu et al. 2019; this study); Guangxi Province, Hechi, Jinchengjiang District (Yu 2010; Hämäläinen 2020).

#### Habitat and biology.

**Figure 9.**
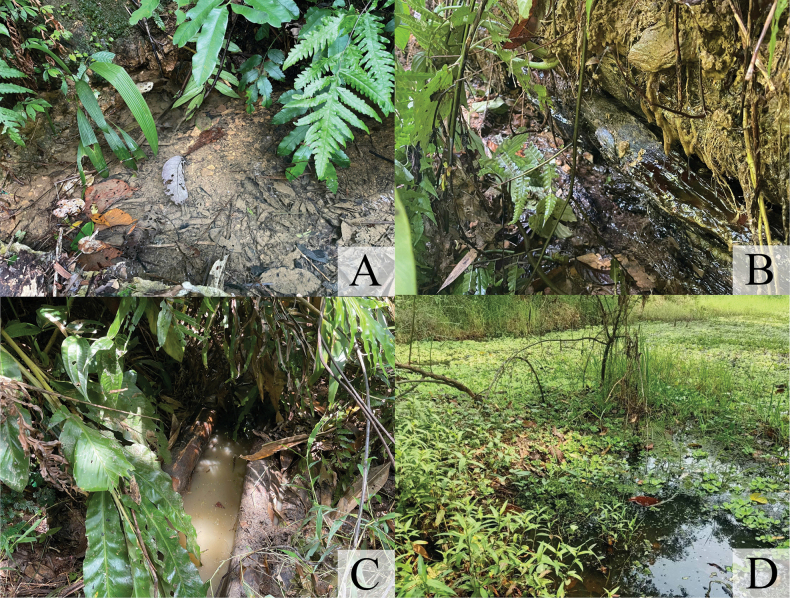
Habitat of *Platycnemisphasmovolans* larva **A** small spring-fed pool in forest **B** rainwater drainage path **C** pool in bamboo trunk (man-made) **D** seasonal forest pool

**Figure 10.**
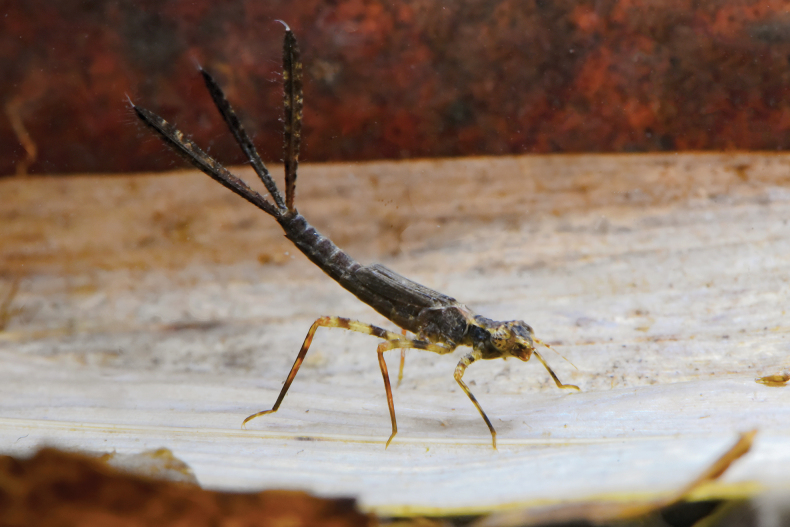
*Platycnemisphasmovolans* larva display agonistic display behaviour.

The larvae of *P.phasmovolans* inhabit forest pools, which are small, shallow bodies of water formed by rainwater or slow-moving streams (Fig. 9). These pools may be temporary or permanent. The larvae secrete themselves among riparian vegetation and leaf litter. The composition of the pool or stream bed where collections were made included silt (30%), small stones/pebbles/gravel/sand (5%), leaf litter (45%), and riparian/root tree debris (20%). The larval coloration acts as camouflage, allowing them to blend perfectly with their surroundings. Black larvae were typically found in dark-brown leaf litter mixed with silt or mud, whereas yellowish/brown larvae were found on riparian roots or brown/yellow leaf litter, such as bamboo leaves. The larvae were found coexisting with the larvae of *Coeliccia* spp., *Coperavittata* (Selys, 1863), and *Coperamarginipes* (Rambur, 1842). Adults of *P.phasmovolans* were also observed mating and laying eggs near the larval habitats. The larvae displayed agonistic behaviour (Fig. 10), characterized by the tendency to hold the distal end of the abdomen slightly upturned, while the caudal lamellae were splayed and pointed upwards.

## ﻿Discussion

This study presents the first description of the larva of *P.phasmovolans*, bringing the total number of documented larval descriptions for *Platycnemis* species from Asia to four: *P.echigoana* by Eda (1965) and Ishida (1996), *P.phyllopoda* by Bae (2011) and Cho (2021), and *P.sasakii* by Ishida (1996). Given the current taxonomic status of *P.phasmovolans*, it is of interest to determine whether the larval stage shares morphological characteristics that reinforce its classification. Examination of larvae of *P.phasmovolans* and *P.phyllopoda* confirmed both share a weakly developed prominence on the anterior occiput – a feature well-developed in congeneric genera such as *Copera*, *Pseudocopera*, and *Spesbona* Dijkstra, 2013 (Deacon and Samways 2016; Saetung et al. 2020). However, this characteristic has been doubted in European species of *Platycnemis*, as it may often be overlooked.

Asian *Platycnemis* species can be distinguished from their European congeners by the presence of four palpal setae on the labial lobe, as opposed to three setae in European species (Ishida 1996; Brochard et al. 2018; this study). Although the number of palpal setae may vary among members of the family Platycnemididae, there is a notable consistency at the species level within the subfamily Platycnemidinae, typically ranging between three and four setae. The larvae of *P.phasmovolans* differ markedly from other Asian congeneric species by having lateral spines on abdominal segments S5–9 (S7–9 in *P.echigoana*, *P.phyllopoda*, and *P.sasakii*) and narrow, lanceolate caudal gills (a broader lanceolate shape in *P.echigoana*, *P.phyllopoda*, and *P.sasakii*).

**Figure 11.**
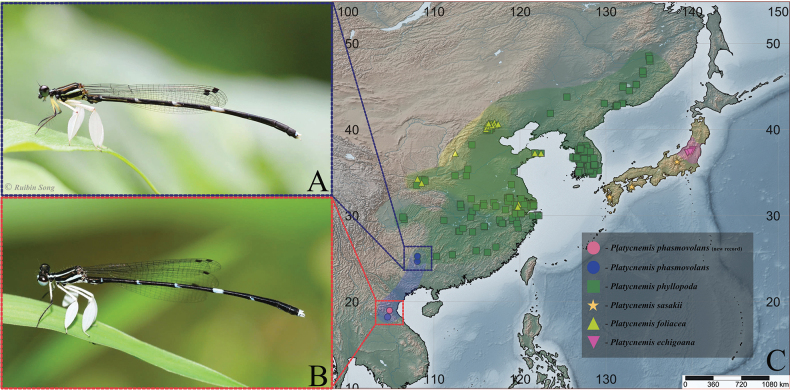
**A** adult *Platycnemisphasmovolans* male from Guizhou Province of China, photo by Rubin Song **B** adult *P.phasmovolans* male from Nghe An Province of Veitnam, photo by Phan Quoc Toan **C** distribution map of genus *Platycnemis*.

The distribution of *Platycnemis* may be restricted by its preference for specific habitats, such as pristine forests and limestone caves (as observed in China). The species appears to be restricted to regions where such conditions occur (Fig. 11C). *Platycnemisphyllopoda* has the broadest distribution, ranging from the southern subtropical regions of Guilin and Zhejiang in China to the northern temperate zones of Jilin, China, and Primorye, Russia (Zhang 2019; Kosterin 2020; iNaturalist 2024d). Regarding *P.hummeli* and *P.ulmifolia*, a thorough examination of the type specimens is necessary to assess whether they are valid synonyms of *P.phyllopoda*. Asahina (1949) originally proposed their synonymy based on observed morphological characters; however, this conclusion was reached without direct examination of the type specimens. Revisiting these types could yield important insights, allowing for verification of Asahina’s taxonomic placement. In contrast, three other species have more restricted ranges: *P.foliacea* occurs in the temperate zone of Beijing and subtropical areas of Xi’an and Shanghai in China (Zhang 2019; Hämäläinen 2021; iNaturalist 2024b), while *P.echigoana* and *P.sasakii* are endemic to Japan (Ishida 1996; Hämäläinen 2021; iNaturalist 2024a, 2024e).

Recent records have expanded the known distribution of *P.phasmovolans* to Vietnam, approximately 80 km from the type locality in Laos (Fig. 11). This finding suggests a broader range within the region, which aligns with previous predictions about the potential distribution of *P.phasmovolans* (Hämäläinen 2003, 2020). On the other hand, there are two earlier records from China (Pu et al. 2019; Hämäläinen 2020). The type locality is located c. 600 km from Nanning and c. 800 km from Maolan (Fig. 11C), raising questions about whether the specimens really are identical to *P.phasmovolans* and necessitating the collection of voucher specimens to confirm their taxonomic status. The Vietnamese and Chinese populations vary in four ways (Chinese population in parentheses): broad stripe on mesepisternum (narrow); presence of white femur on fore legs (pale yellow); marking on S6 0.35 times as long as S6 length (0.40); and presence of distinct baso-ventral stripe on S7 (ambiguous) (Fig. 11A, B). This study does not attempt to resolve the taxonomic uncertainties surrounding *Platycnemis* but aims to provide data and hypotheses that may support future analyses. The comparisons made here may offer insights into the evolutionary position of *Platycnemis* within its genus based on larval characteristics.

Currently, data on *P.phasmovolans* is significantly lacking, leading to its classification as “Data Deficient” by the IUCN (Hämäläinen 2011). GBIF (2024) provides information on the type material only. There are no records of *P.phasmovolans* available in iNaturalist (2024c), which moreover includes a misidentified photograph without any information about the source. Given these discrepancies and the possibility of undiscovered populations, intensive surveys in unexplored areas, particularly in northern Vietnam, the upper parts of Laos, and southern to southwestern China, are highly recommended. Such efforts are crucial to developing a comprehensive understanding of the distribution and ecological requirements of *P.phasmovolans* and its congeneric species across the Sino-Japanese and Indo-China regions.

### ﻿Key to the known larvae of Platycnemidinae in the Sino-Japanese and Oriental regions

The subfamily Platycnemidinae in the Sino-Japanese and Oriental regions includes 16 species in two tribes: Coperini (*Copera*, 5 species) and Platycnemidini (*Matticnemis*, 1 species; *Platycnemis*, 6 species; and *Pseudocopera*, 4 species) (Ishida 1996; Zhang 2019; Saetung et al. 2020; Kalkman et al. 2020; Cho 2021; Dow et al. 2024). The larvae of 10 species have been described based on studies by Eda (1965), Ishida (1996), Yum and Bae (2007), Bae (2011), Saetung et al. (2020), Cho (2021), and this study. This subfamily is recognized by the presence of raised prominences on the occipital margins behind the eyes and the presence of two pairs of premental setae.

**Table:** 

1	Caudal lamellae with frilled borders	**2 (Coperini: *Copera*)**
–	Caudal lamellae with elongate and smooth borders	**4 (Platycnemidini)**
2	Number of fringe filaments less than 20; short, fringe filaments stout basally; sometimes poorly fimbriated	***C.chantaburii*** [Indochina region]
–	Number of fringe filaments more than 20; long, fringe filaments stout or narrow basally	**3**
3	Slender, fringe filaments stout basally; spiky, jagged appearance (Fig. 12A)	***C.marginipes*** [Oriental region]
–	Hair-like, slightly curved fringes, very narrow basally; wavy appearance (Fig. 12B)	***C.vittata*** [Oriental region]
4	Palpal lobe with three palpal setae; moderately produced ligula; well-developed protuberance on occipital margin; body shorter than 1.3 × length of caudal gills	**5 (*Pseudocopera*)**
–	Palpal lobe with four palpal setae; strongly produced ligula; poorly-developed protuberance on occipital margin; body at least as long as 1.5 × length of caudal lamellae	**7 (*Platycnemis*)**
5	S8–9 without lateral spines	***Ps.rubripes*** [Sino-Japan region]
–	S8–9 or S9 with lateral spines	**6**
6	Caudal lamellae length longer than 0.8× body length; with one pair seta on the terminal filament of the caudal lamellae; with lateral spine on S9	***Ps.ciliata*** [Oriental region]
–	Caudal lamellae length shorter than 0.8× body length; without one pair seta on the terminal filament of the caudal lamellae; with lateral spine on S8–9	***Ps.annulata*** [Sino-Japan region]
7	S5–9 with lateral spines	***Pl.phasmovolans*** [Indochina region]
–	S7–9 with lateral spines	**8**
8	Postocular lobe rounded; shallow posterior lobes	***Pl.sasakii*** [Sino-Japan region]
–	Postocular lobe angulated; deep posterior lobes	**9**
9	Ligula with small median cleft; terminal filaments on apex of caudal gills long	***Pl.echigoana*** [Sino-Japan region]
–	Ligula without median cleft; terminal filaments of caudal gills short or absent	***Pl.phyllopoda*** [Sino-Japan region]

**Figure 12.**
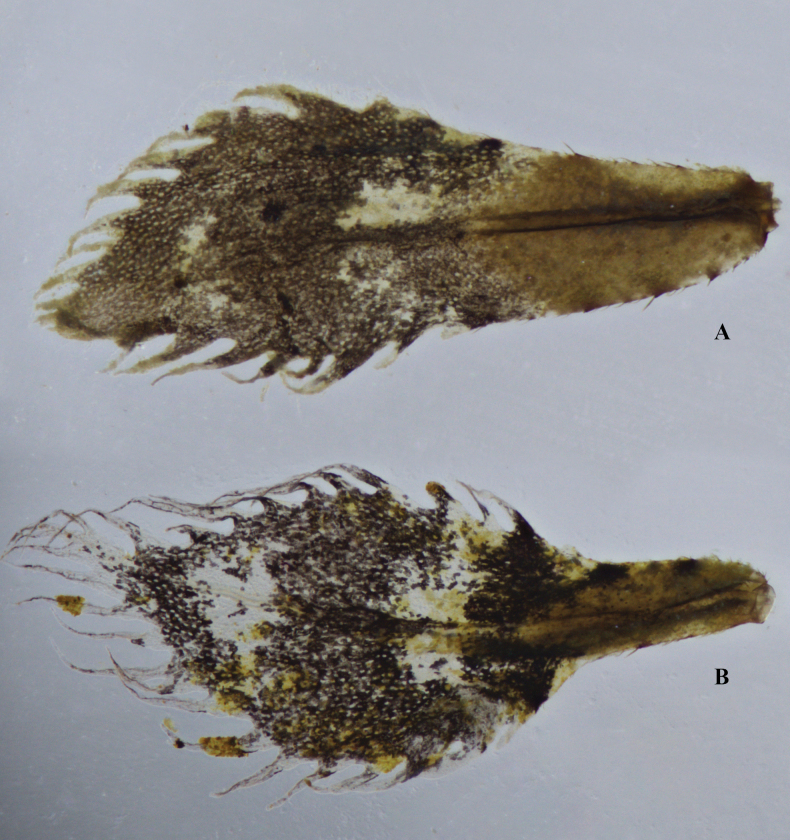
Caudal gills of *Copera* spp. **A***C.marginipes***B***C.vittata*.

## Supplementary Material

XML Treatment for Platycnemis phasmovolans

## ﻿Additional information

### Author ORCIDs

Tosaphol Saetung Keetapithchayakul https://orcid.org/0000-0001-7565-4701

Quoc Toan Phan https://orcid.org/0000-0002-3154-6546

### Data availability

All of the data that support the findings of this study are available in the main text.
